# Supplementary material for: SyNDI: synchronous network data integration framework
Source: BMC Bioinformatics. 2018 Nov 6;19:403. doi: 10.1186/s12859-018-2426-5 (PMC6219086; doi:10.1186/s12859-018-2426-5)
Supplement: Supplementary file 5 — Files of the example presented in Additional file 4. This additional file is a zip package that contains all files used in Additional file 4. Detailed descriptions of each of these files are presented in a README file included in this zip package. (GZ 30411 kb) [file 12859_2018_2426_MOESM5_ESM.gz › SyNDITools/Meme2Fimo/meme_4.9.0_4.tar.gz!/meme_4.9.0/doc/gomo-format.html]

GOMO file Format


# GOMO file format

GOMO outputs an xml file using the following format.

| Tag | Child of | Description |
| --- | --- | --- |
| <gomo> | Nothing | Information about this run of gomo.  - version - The version of gomo that generated the xml file. - release - The release date of the version that generated the xml. |
| <program> | <gomo> | Information about the state of the program when it ran.   - name - name of the program. - cmd - the command line passed to the program. - gene\_url - the url used to lookup further information on the gene ids.   The url has ampersands (&) converted into **&amp;** and the place where   the gene ID should be replaced by **!!GENEID!!** . - outdir - the output directory that the program wrote to. - clobber - true if gomo was allowed to overwrite the output directory. - text\_only - true if gomo wrote to stdout, in which case this file would   not exist so it must be false. - use\_e\_values - true if gomo used E-values (converted from p-values) as   input scores, false if gomo used gene scores. - score\_e\_thresh - if gomo used E-values then this is the threshold that   gomo assumed the worst E-value (p-value = 1.0) for the gene to smooth out noise. - min\_gene\_count - the minimum number of genes that a GO term was annotated   with before gomo would calculate a score for it. - motifs - if present then a space delimited list of the motifs that gomo   calculated a score for, othewise gomo scored all motifs. - shuffle\_scores - the number of times gomo generated a shuffled mapping of   gene id to gene id to be used to generate scores from the null model. - q\_threshold - gomo filtered the results to only show those with a better   (smaller) q-value. |
| <gomapfile> | <program> | Information about the GO mapping file.   - path - the path to the mapping file. |
| <seqscorefile> | <program> | Information about the sequence scoring file.   - path - the path to the sequence scoring file. |
| <motif> | <gomo> | Information about the motif.   - id - the motif identifier. - genecount - the number of scored sequences that were used to compute the result. |
| <goterm> | <motif> | Information about the GO term.   - id - the GO identifier. - score - the geometic mean across all species of the rank-sum test p-value. - pvalue - the empirically calculated p-value. - qvalue - the empirically calculated q-value. - annotated - the number of genes annotated with the go term. - group - the subgroup that the term belongs to. For the Gene Ontology   b = biological process, c = cellular component and m = molecular function. - nabove - the number of more general terms that link to this one. - nbelow - the number of more specific terms that link from this one. - implied - is the go term implied by other significant go terms?   Allows values 'y', 'n' or 'u' (default) for yes, no or unknown. - description - the GO term description. |
| <gene> | <goterm> | Information about the GO term's annotated genes for the primary species.   - id - the gene identifier. - rank - the rank of the scored gene. |
